# CHINESE–WHITE DIFFERENCES IN SOCIAL ACTIVITY, SOCIAL SUPPORT, AND MENTAL HEALTH AMONG OLDER ADULTS

**DOI:** 10.1093/geroni/igae098.3428

**Published:** 2024-12-31

**Authors:** Lingming Chen, Elizabeth Procter-Gray, Qun Le, Danielle LoPilato, Marianella Ferretto, Meng Zhang, Kevin Kane, Wenjun Li

**Affiliations:** University of Massachusetts Lowell, Lowell, Massachusetts, United States; University of Massachusetts Lowell, Lowell, Massachusetts, United States; University of Massachusetts Lowell, Lowell, Massachusetts, United States; University of Massachusetts Lowell, Lowell, Massachusetts, United States; University of Massachusetts Lowell, Lowell, Massachusetts, United States; University of Massachusetts Lowell, Lowell, Massachusetts, United States; University of Massachusetts Lowell, Lowell, Massachusetts, United States; University of Massachusetts Lowell, Lowell, Massachusetts, United States

## Abstract

Studies on healthy aging of older Asian Americans, especially Chinese Americans, are rare. This study evaluated Chinese – Non-Hispanic White differences in social activity, social support and mental health. The study enrolled 554 community-dwelling older adults (434 Non-Hispanic White and 120 Chinese) aged 65-95 years old in central and northeastern Massachusetts (2018-2023). Social activity and social support were measured by the Lifestyle Questionnaire. Mental health includes depressive symptoms assessed by the Center for Epidemiologic Studies Depression Scale, anxiety symptoms assessed by the Beck Anxiety Inventory, perceived stress assessed by the Perceived Stress Scale, and resilience assessed by the modified Brief Resilience Scale. Chinese - White differences in these factors were analyzed using linear regression models adjusting for age, gender, self-rated health, physical activity, body pain, and living alone. Results showed that in both unadjusted and adjusted models, older Chinese had less social support, lower frequency of social activities, and lower level of resilience compared to Non-Hispanic Whites. In the unadjusted models, older Chinese had higher levels of perceived stress, anxiety, and depressive symptoms compared to Non-Hispanic Whites, but there were not differences in these three factors after adjusting for covariables. In conclusion, social activity, social support, and mental health were different between Chinese and Non-Hispanic Whites among older adults in the study population. The findings from this study can inform the design and implementation of health programs tailored to specific groups, thereby reducing health disparities and improving overall well-being for older adults.

